# *In Vitro* Evaluation of Antimicrobial Activity and Cytotoxicity of Different Nanobiotics Targeting Multidrug Resistant and Biofilm Forming Staphylococci

**DOI:** 10.1155/2018/7658238

**Published:** 2018-11-28

**Authors:** Mennatallah A. Mohamed, Maha Nasr, Walid F. Elkhatib, Wafaa N. Eltayeb

**Affiliations:** ^1^Microbiology Department, Faculty of Pharmacy, Misr International University, Cairo 19648, Egypt; ^2^Pharmaceutics and Industrial Pharmacy Department, Faculty of Pharmacy, Ain Shams University, African Union Organization St., Abbassia, Cairo 11566, Egypt; ^3^Pharmaceutics and Pharmaceutical Technology Department, Faculty of Pharmacy, Mutah University, Mutah, Jordan; ^4^Microbiology and Immunology Department, Faculty of Pharmacy, Ain Shams University, African Union Organization St., Abbassia, Cairo 11566, Egypt; ^5^Department of Microbiology and Immunology, School of Pharmacy & Pharmaceutical Industries, Badr University in Cairo (BUC), Entertainment Area, Badr City, Cairo, Egypt

## Abstract

Antibiotic-resistant and biofilm-forming bacteria have surprisingly increased over recent years. On the contrary, the rate of development of new antibiotics to treat these emerging superbugs is very slow. Therefore, the aim of this study was to prepare novel nanobiotic formulations to improve the antimicrobial activity of three antibiotics (linezolid, doxycycline, and clindamycin) against* Staphylococci*. Antibiotics were formulated as nanoemulsions and evaluated for their antimicrobial activities and cytotoxicities. Cytotoxicity of the conventional antibiotics and nanobiotics was analyzed using 3-(4,5-dimethylthiazol-2-yl)-2,5-diphenyltetrazolium bromide (MTT) assay on rat hepatocytes. Half-maximal inhibitory concentration (IC_50_) was estimated from an experimentally derived dose-response curve for each concentration using GraphPad Prism software. Upon quantitative assessment of* Staphylococcus *biofilm formation, eighty-four isolates (66.14 %) were biofilm forming. Linezolid and doxycycline nanobiotics exhibited promising antibacterial activities. On the contrary, clindamycin nanobiotic exhibited poor antibacterial activity. Minimum biofilm inhibitory concentrations showed that 73.68 %, 45.6%, and 5.2% of isolates were sensitive to linezolid, doxycycline, and clindamycin nanobiotics, respectively. Results of this study revealed that antibiotics loaded in nanosystems had a higher antimicrobial activity and lower cytotoxicities as compared to those of conventional free antibiotics, indicating their potential therapeutic values.

## 1. Introduction

Bacterial infections are the second acknowledged cause of death worldwide and the third in developed countries. The therapeutic efficiency of antimicrobials has become more complex due to the emergence of multidrug resistance [1–3].* Staphylococcus *is perhaps the pathogen of greatest concern because of its intrinsic virulence, ability to cause many life-threatening infections, its capacity to adapt to different environmental conditions, and biofilm formation [4, 5].

A promising approach for the management of emerging bacterial resistance is antibiotic formulation in nanostructures with dimensions of approximately 1 to 100 nm [3, 6, 7]. The newly designed formulations are known as nanoantibiotics or nanobiotics. Such nanobiotics offer many distinctive advantages when compared to conventional antibiotics such as improved solubility of drugs, prolonged drug half-life, and systemic circulation time which accordingly lowers administration frequency and dose [8–11].

Oil in water (O/W) nanoemulsions are thermodynamically stable solutions and easy to prepare and can solubilize poorly soluble drugs [12–14]. Antibiotic-nanoemulsion decreases cell toxicity by facilitating the binding of antibiotics to bacteria, increasing the concentration of antibiotics at the site of bacterium-antibiotic interaction and hence decreasing the requirement for high doses [14, 15]. Nanoemulsions of antibiotics may serve as a promising alternative to standard antibiotic formulations to enhance antimicrobial properties as well as anti-biofilm activities of antibiotics [16, 17].

In the present study, novel nanobiotics were formulated and further evaluated for their antimicrobial activities and cytotoxicities in comparison with the conventional antibiotics.

## 2. Materials and Methods 

### 2.1. Bacterial Isolates and Antimicrobial Agents

A total of 127 staphylococcal isolates were recovered from 200 clinical blood specimens (Ain Shams University Hospitals, Cairo, Egypt). Isolates were identified as described in Bergy's manual of determinative bacteriology, 9^th^ edition [18]. The standard strain used in the present study was* S. aureus *ATCC 25923. Clindamycin and linezolid antibiotics were provided by Egyptian group for pharmaceutical industries, El Obour, Cairo, Egypt. Doxycycline was provided by EIPICO, 10^th^ of Ramadan City, Cairo, Egypt. Stock solutions from dry powders were prepared at a concentration of 2560 *µ*g/ml for the antibiotics and preserved at - 20°C.

### 2.2. Biofilm Formation Assay

Overnight cultures grown in tryptic soy broth (TSB; Oxoid, England) at 37°C were diluted in sterile TSB to match 0.5 McFarland standard equivalent to 1.5 × 10^8^ CFU/ml. These bacterial suspensions were further diluted 1:100 in TSB supplemented with 2% w/v glucose and 2% w/v sodium chloride. Two hundred microliters of these suspensions was aseptically transferred to each of three parallel wells of a 96-well, nontreated polystyrene microtiter plate (Corning, NY, USA). After incubation at 37°C for 24 h, the absorbance at wavelength 562 nm was recorded using Biochrom ASYS expert plus microplate reader (Biochrom, MA, USA) as a measure of total growth. Furthermore, the culture was removed and plates were carefully rinsed three times with 200 *μ*l of tryptone water (Sigma-Aldrich, St. Louis, MO, USA) to remove nonadherent cells and were subsequently air-dried at room temperature. The established biofilms were stained with 100 *μ*l/well of 0.1% membrane filtered crystal violet solution (Sigma-Aldrich, St. Louis, MO, USA) at room temperature for 2 min. Crystal violet solution was removed and the biofilms were washed twice with 200 *μ*l phosphate buffered saline (Sigma-Aldrich, St. Louis, MO, USA). In order to elute the bound crystal violet, 100 *μ*l of a mixture of 80% ethanol and 20% acetone was introduced to each well and the plate was then incubated for 20 mins at room temperature. Finally, the eluted crystal violet was diluted in a new plate with ethanol/acetone mixture (ratio = 1:10) and the optical density was measured at *λ*_562_ nm as described previously by Elkhatib* et al.*, 2014 [19].

### 2.3. Determination of Minimum Biofilm Inhibitory Concentration (MBIC)

Seventy-five-microliter inoculums of 1.5x 10^8^ CFU/ml TSB culture were incubated for 24 h at 37°C in polystyrene, round bottom 96-well microplates (Corning, NY, USA). After incubation, the supernatant was aspirated and the wells were washed twice with sterile normal saline solution. Then, one hundred microliters of twofold serial dilutions of antibiotics and nanobiotics, in cation-adjusted Mueller Hinton II broth (Oxoid, Basingstoke, UK), was added to the wells with the established biofilms. After incubation for 18 h at 37°C, MBIC was recorded. MBIC is defined as the lowest concentration of the antibiotic/nanobiotic that results in no visible growth [20].

### 2.4. Preparation and Characterization of the Antibiotic Loaded Nanoemulsions

The plain and antibiotic loaded nanoemulsions (nanobiotics) were prepared as described previously [21–24]. Briefly, each tested antibiotic (clindamycin, linezolid, and doxycycline) was incorporated in 4.1 ml tween 20, 0.28 ml oleic acid, and 0.32 ml ethanol and stirred by a magnetic stirrer (Yellow line MAG HS7, IKA, France). Then, the mixture was titrated with 5.6 ml water dropwise for formulation of oil in water nanoemulsion. The nanoemulsion was prepared at a concentration of 128 *µ*g/ml of each antibiotic. The particle size, zeta potential, and polydispersity index (PDI) of the prepared nanoemulsions were measured using the Zetasizer device (model ZS3600, Malvern, UK).

### 2.5. Cytotoxicity Assessment on Rat Hepatocytes

#### 2.5.1. Rat Hepatocyte Isolation and Cell Culture

Sprague-Dawley male rats (200-250 g) were obtained from the animal's house, Faculty of Science, Al-Azhar University, Cairo, Egypt. Hepatocytes were isolated according to the collagenase perfusion procedure as described formerly [25]. Hepatocytes (1x10^6^ cells/ml) were placed into Krebs-Henseleit buffer (pH 7.4) containing 12.5 mM HEPES (Sigma-Aldrich, St. Louis, MO, USA) and maintained at 37°C with 95% O_2_ and 5% CO_2_. Hepatocytes with viability more than 90% were used in the experiments. The cells were grown on RPMI-1640 medium (Lonza, Bornem, Belgium) supplemented with 10% v/v inactivated fetal calf serum (Lonza, Bornem, Belgium) and 50 *µ*g/ml gentamicin (Lonza, Bornem, Belgium). The cells were maintained at 37°C in a humidified atmosphere with 5% CO_2_ and were subcultured two to three times a week [26].

#### 2.5.2. Cytotoxicity Evaluation Using 3-(4,5-Dimethylthiazol-2-yl)-2,5-diphenyltetrazolium Bromide (MTT) Cell Viability Assay

Hepatocytes were seeded in 96-well tissue culture plates (Corning, NY, USA) at concentration of 2x10^6^ cells/well. After 24 h incubation at 37°C, six concentrations (2, 4, 8, 16, 32, and 64 *µ*g/ml) of the nanobiotics (clindamycin, linezolid, and doxycycline) and the conventional antibiotics were added to the wells containing cells in triplicate with control and blank in each plate. After 24 h, the number of viable cells was determined as described previously [27]. Briefly, the tissue culture medium was removed from the 96-well plate and replaced with 100 *µ*l of fresh RPMI 1640 medium without phenol red and then 10 *µ*l of the 12 mM MTT (Sigma-Aldrich, St. Louis, MO, USA) stock solution (5 mg of MTT in 1 ml of PBS) was added to each well including the untreated controls. The 96-well plates were then incubated at 37°C and 5% CO_2_ for 4 h. An aliquot (85 *µ*l) of the medium was removed from the wells, and 50 *µ*l of dimethyl sulfoxide (Sigma-Aldrich, St. Louis, MO, USA) was added to each well and mixed thoroughly with the pipette and incubated at 37°C for 10 min. The optical density was measured at 590 nm with SunRise™ microplate reader (TECAN, Männedorf, Switzerland) to determine the number of viable cells based on the selective ability of viable cells to reduce the tetrazolium component of MTT into purple colored formazan crystals. The 50% drug inhibitory concentration (IC_50_), drug concentration that reduces the viability of the intact cells by 50%, was estimated from the graphic plot of the dose response curve for each concentration using GraphPad Prism version 7.0 for Windows (GraphPad Software, La Jolla, CA, USA) [27].

## 3. Results

### 3.1. Staphylococci Identification and Biofilm Formation Assay

*Staphylococcus *isolates (n = 127) were identified as shown in Table 1 and quantitatively analyzed for biofilm formation. The isolates were categorized into three groups according to the optical density: strong biofilm forming (OD_562_> 1.11), weak biofilm forming (OD_562_ 0.22–1.11), and non-biofilm forming (OD_562_ < 0.22) as we described previously [19]. Of the 127 isolates, 57 (44.88%) showed strong biofilm-forming ability, 27 (21.26%) showed weak biofilm ability, and 43 (33.85%) showed no biofilm formation. Strong biofilm-formers were* Staphylococcus aureus *(n = 31)*, S. epidermidis *(n = 21)*, S. haemolyticus *(n = 2), and* S. lugdunensis *(n = 3).

### 3.2. Determination of Minimum Biofilm Inhibitory Concentration (MBIC)

Strong biofilm-formers were selected for evaluation of the antimicrobial activity of the free antibiotics as well as the formulated nanobiotics. The results revealed that all biofilms of the tested isolates were resistant to clindamycin (MICs≥ 4 *μ*g/ml), doxycycline (MICs≥ 16 *μ*g/ml), and linezolid (MICs≥ 8 *μ*g/ml) according to CLSI (2017) guidelines [28]. Among the tested* S. aureus, *87.09% and 25.8% were sensitive to linezolid and doxycycline nanobiotics, respectively, while 41.9% and 6.45% were intermediately resistant to doxycycline and clindamycin nanobiotics, respectively. On the other hand,* S. epidermidis* showed 57.14% sensitivity to linezolid nanobiotic, while 28.57% and 4.76% were intermediately resistant towards doxycycline and clindamycin nanobiotics, respectively.* S. lugdunensis *and* S. haemolyticus *showed 100% sensitivity to linezolid nanobiotic, while both showed 100% resistance to doxycycline and clindamycin nanobiotics.  Table 2 represents the MBICs of the three nanobiotics against* S. aureus*,* S. epidermidis, S. lugdunensis, *and* S. haemolyticus.*

### 3.3. Characterization of the Prepared Nanoemulsions

The nanoemulsions were successfully prepared at very small nanometer size ranging from 10.66 to 13.93 nm, zeta potential values ranging from -9.95 to -12.7 mV, and polydispersity indices ranging from 0.29 to 0.38, indicating homogenously prepared disperse systems. Results of nanoemulsion characterization are shown in Table 3.

### 3.4. Cytotoxicity Evaluation Using MTT Cell Viability Assay

**Figure 1:**
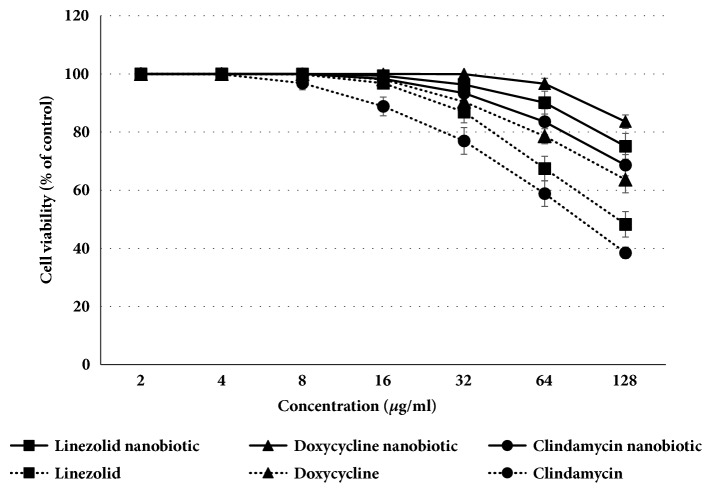
Cell viability of rat hepatocytes presented as percentage of control after 24 h exposure to different concentrations of linezolid, doxycycline, and clindamycin nanobiotics as compared to their conventional antibiotics. The data represent the average values of three experiments (± SD).

Cell viability assay (Figure 1) verified that hepatocytes exposed to nanobiotic formulations revealed higher percentages of viable cells as compared to that of their conventional antibiotics proving that nanobiotics are less toxic and more cyto-compatible. The MTT assay revealed that hepatocytes viability was significantly (P<0.05) higher by 16.47-30.15%,9.49-26.83%, and 8.11-14.57% upon exposure to clindamycin, linezolid, and doxycycline nanobiotics, respectively, as compared to that of their free antibiotics at concentration ranges of 16 to 64 *µ*g/ml. Linezolid, doxycycline, and clindamycin nanobiotics showed weak inhibitory activity against rat hepatocytes with IC_50_> 64 *µ*g/ml and inhibitory activity of 61.1±6.18*µ*g/ml, > 64 *µ*g/ml, and 45.8±2.45 *µ*g/ml for linezolid, doxycycline, and clindamycin antibiotics, respectively.

## 4. Discussion

Microbial biofilm represents a major virulence factor associated with chronic and recurrent infections [29–32]. Biofilm formation is the key component of the lack of efficacy of standard antimicrobials against* Staphylococci* as it serves to protect the bacteria, thus preventing the penetration of antimicrobial agents [33]. An emerging approach to face the problem of antimicrobial resistance can be done by encapsulating the antibiotic in a stable nanosystem to improve the drug delivery and localize the drug release at the site of action to decrease the side effects [34, 35].

In the present study, nanoemulsion formulations were used for the encapsulation of three antibiotics including linezolid, doxycycline, and clindamycin, because they are thermodynamically stable solutions and easy to prepare and can solubilize poorly soluble drugs, thus enhancing their bioavailability [14, 36–38].

The selection of the tested antimicrobial agents was based on the proven efficacy and acceptable* in vitro *test results reported by CLSI (2017) [28]. Clindamycin is a member of group A antimicrobial agents in CLSI which is considered to be the most appropriate for inclusion in routine and primary testing panel against* Staphylococci*. Doxycycline and linezolid, members of group B antimicrobial agents, which are therapeutically used in case of tolerance, fail to respond to any antimicrobial agent in group A, or when the microorganism is resistant to agents of the same antimicrobial class [28].

Antimicrobial susceptibility against biofilms of the tested* Staphylococci* revealed that all isolates were resistant to the conventional linezolid antibiotic, and this striking resistance could be attributed to mutations to the central loop of Domain V of the 23S rRNA, which lies in the 50S ribosomal subunit, and these mutations presumably alter linezolid's binding site [39]. However, 73.68% of the tested isolates were sensitive to linezolid nanobiotic. These results were in agreement with those previously reported by Hedaya* et al.* (2017) [40] stating that the oral bioavailability of linezolid administered as suspension was 38.7% and has increased significantly when administered as nanoemulsion to 51.7%. According to the biopharmaceutical classification system (BCS), linezolid has been classified as a Class IV drug; this classification was based on being lipophilic with low solubility and permeability across the gastrointestinal membrane. Improved linezolid nanoemulsion bioactivity from 38.7% to 51.7% could be attributed to improved water solubility that has resulted in improved systemic bioavailability and, possibly, the* in vivo* antibacterial activity [40].

Doxycycline nanobiotic was found to be active against 45.6% of the tested* Staphylococcus* isolates. Comparable results were reported by Narang* et al. *(2015) [41], as tetracycline nanoemulsion exhibited antibacterial activity against S. aureus and was found to be effective in reduction of the bacterial load at the site of infection. Our results agreed with those reported by Maya* et al*. (2012) [42] who mentioned that encapsulated tetracyclines showed sustained release and improved the bioavailability of the drug. Moreover, specific binding of nanoparticles with* S. aureus* indicated its potential use for targeted delivery against serious* S. aureus *infections including sepsis, endocarditis, and pneumonia, especially, with biofilm-associated infections.

On the other hand, clindamycin could not show a great enhanced antibacterial activity against the tested* Staphylococci* with only 5.2% of isolates changed from resistant to sensitive. This might be explained by the fact that all the clinical isolates used in the study were resistant to clindamycin from the beginning, and drug encapsulated nanoemulsions can be used for enhancement of antibacterial activity only, if already present [14], and possibly our formulated clindamycin nanobiotic could not effectively penetrate the matrix of the tested staphylococcal biofilms for reaching their cellular targets. However, Prasad* et al.* (2012) [43] reported that a gel formulation of clindamycin, prepared as nanoemulsion for topical application, was more effective in improving and alleviating both inflammatory and noninflammatory skin lesions than the conventional clindamycin.* In vitro* and clinical studies have shown that the topical nanoformulation can improve the penetration of active ingredients into the epidermis and dermis which make clindamycin conjugated nanoemulsion very effective when used topically [43].

There are challenges facing the application of this strategy for clinical use including the interaction of nanobiotics with cells, tissues, and organs [34, 44]. Theoretically, nanoparticles are retained much longer in the body than antibiotics, which could be beneficial for achieving sustained therapeutic effects [45, 46]. On the other hand, the safety profiles of nanosized drug carriers, especially upon long-term exposure, should be considered because there are some concerns regarding safety [47–50]. Due to this challenge, “nanotoxicology” term was adopted and defined as the science dealing with the effects of nanostructures on living organisms [51].

Cell based assays are often used for screening of novel formulations to determine if the test molecules are having direct cytotoxic effects. MTT assay was used in the present study; viable cells with active metabolism have the ability to convert MTT into a purple colored formazan with a maximum absorbance at 590 nm. When cells die, they lose the ability to convert MTT into formazan; thus color formation is the marker of only the viable cells [52, 53].

Cytotoxicity results demonstrated that linezolid, doxycycline, and clindamycin nanobiotics had better safety profiles than those of the conventional antibiotics. Our results agreed with Tariq* et al. *(2016) [14] mentioning that antibiotics in nanoformulation could decrease cell toxicity by facilitating the binding of antibiotics to bacteria, increasing the concentration of antibiotics at the site of bacterium-antibiotic interaction; consequently, such nanoformulations may limit the requirement for high doses in case of serious infections. Similarly, it has been proved by Jiyauddin* et al* (2015) [54] that, with the help of nanoemulsion as a delivery system, retention time of a drug in the body can be increased, so lower concentration of drug may be required for achieving the same therapeutic outcome.

## 5. Conclusions

Advances in nanotechnology have facilitated the design of new nanoantibiotics for various pharmaceutical and therapeutic applications to counteract the global public health threats resulting from antimicrobial-resistant pathogens. Microbial resistance, especially with biofilm formation, can be eliminated by formulating antibiotics into nanoparticles. Loading antibiotics on nanostructures possesses several clinical advantages allowing them to overcome solubility and stability issues of conventional antibiotics and minimizes drug-induced side effects. Furthermore, antibiotic-loaded nanostructures ensure high local concentrations of therapeutic molecules at their target sites, improve antibacterial properties, and decrease the drug toxicity as compared to that of the free antibiotics. In conclusion, nanotechnology can lead to a breakthrough in the development of various nanobiotics and nanoantimicrobial agents, which may reduce the public health threats from recalcitrant infectious diseases and biofilm-associated infections.

## Figures and Tables

**Table 1:** Identification of *Staphylococcus* clinical isolates.

*Staphylococcus* species (n = 127)	*S. aureus*	*S.*	*S. lugdunensis*	*S.*	*S.*
		*epidermidis*		*haemolyticus*	*intermedius*
Number of isolates (%)	63 (49.6)	44 (34.65)	9 (7.1)	7 (5.5)	4 (3.15)

**Table 2:** Minimum biofilm inhibitory concentrations (MBICs) of linezolid, doxycycline, and clindamycin nanobiotics against biofilms of different species of *Staphylococci*.

Species (No. of Isolates)	MBIC (*μ*g/ml) of			MBIC (*μ*g/ml) of			MBIC (*μ*g/ml) of		
	Linezolid			Doxycycline			Clindamycin		
	S (%)	`I (%)	R (%)	S (%)	I (%)	R (%)	S (%)	I (%)	R (%)
	≤ 4	-	≥ 8	≤ 4	8	≥ 16	≤ 0.5	1-2	≥ 4
*S. aureus* (n=31)	27 (87.09)	-	4 (12.9)	8 (25.8)	13 (41.9)	10 (32.25)	-	2(6.45)	29 (93.54)
*S. epidermidis* (n=21)	12 (57.14)	-	9 (42.86)	2 (9.52)	6 (28.57)	13 (61.9)	-	1 (4.76)	20 (95.24)
*S. haemolyticus* (n=2)	2 (100)	-	-	-	-	2 (100)	-	-	2 (100)
*S. lugdunensis* (n=3)	3 (100)	-	-	-	-	3 (100)	-	-	3 (100)

R= resistant; I= intermediate resistance; S= sensitive. The cutoff values proposed by the CLSI (2017) of MBIC (*μ*g/ml): for linezolid S ≤ 4, R ≥ 8, for doxycycline S≤ 4, I=8, R ≥ 16, and for clindamycin S ≤ 0.5, I =1-2, R ≥ 4.

**Table 3:** Characterization of the prepared nanoemulsions.

Formulation	Particle size	Zeta potential	PDI
	(nm)	(mV)	
Clindamycin nanoemulsion	13.93±0.62	-12.70±3.11	0.38±0.06
Linezolid nanoemulsion	11.52±0.96	-10.40±1.65	0.32±0.02
Doxycycline nanoemulsion	10.66±0.11	-9.95±0.77	0.29±0.03

## Data Availability

The “antimicrobial activity and cytotoxicity of different nanobiotics” data used to support the findings of this study are included within the article.
